# Clinical characteristics and risk factors for overlapping rheumatoid arthritis and Sjögren’s syndrome

**DOI:** 10.1038/s41598-018-24279-1

**Published:** 2018-04-18

**Authors:** Huaxia Yang, Sainan Bian, Hua Chen, Li Wang, Lidan Zhao, Xuan Zhang, Yan Zhao, Xiaofeng Zeng, Fengchun Zhang

**Affiliations:** 1 Department of Rheumatology and Clinical Immunology, Peking Union Medical College Hospital, Chinese Academy of Medical Sciences, Dongcheng, China; 2 The Ministry of Education Key Laboratory, Peking Union Medical College Hospital, Chinese Academy of Medical Sciences, Dongcheng, China

## Abstract

This study investigated the clinical characteristics and risk factors for overlapping rheumatoid arthritis and Sjögren’s syndrome (RA/SS). Patients with RA/SS in Peking Union Medical College Hospital from January 2012 to January 2017 were retrospectively analysed and compared to those of sex- and age–matched RA or SS controls. Logistic regression analysis was used to identify risk factors. Altogether, 105 consecutive patients with RA/SS were enrolled. Ninety-seven (92.4%) of them were female, with a mean age of 51.5 ± 13.3 years or 45.2 ± 14.7years at the diagnosis of SS or RA, respectively. In addition to arthritis and Sicca symptom, patients with RA/SS had more visceral involvements including interstitial lung disease (ILD), and haematologic involvement, and received more glucocorticoid treatments than controls (p < 0.05). RA-onset, simultaneous-onset and SS-onset patients had significant differences in age at RA diagnosis, fever and thrombocytopenia (p < 0.05). Multivariate logistic analysis indicated that arthritis (OR = 44.804), rheumatoid factor (RF) (OR = 5.973), and anti-CCP (OR = 2.545) were independent risk factors for SS overlapping with RA. Xerostomia (OR = 3.960), ILD (OR = 6.210), and anti-SSA (OR = 24.640) were independent predictors of RA overlapping with SS. RA/SS patients have more visceral involvements. Our findings highlight the roles of arthritis/RF/anti-CCP and xerostomia/ILD/anti-SSA in the development of this overlapping disease.

## Introduction

Sjögren syndrome (SS) is a progressive autoimmune disease with the hallmark manifestation of Sicca symptom and systemic manifestations^1^. Joint lesions, although rare, are suggestive of Sjögren syndrome. Positive rheumatoid factor (RF) is sometimes accompanied by the joint signs in SS patients^2^. Rheumatoid arthritis (RA) is characterized by its clinical manifestations of joint inflammation, bone erosion and serologic autoantibodies to citrullinated protein antigens (ACPAs) and RF^2^. Previous studies on the overlap of RA and SS (RA/SS) are limited. The association between SS and RA is unclear.

In general, while SS can occur in combination with another autoimmune disease, the accompanying disease is the main determinant of the phenotype and treatment^3^. However, when consistent with RA, the SS patients had distinctive phenotypes other than joint involvements. He *et al*. noted its predilection for more complications and systemic involvement^4–7^. However, the risk factors for RA/SS have not been identified. Moreover, most SS occurs after RA, whereas some RA and SS occur simultaneously or RA onset occurs after SS. The chronologic order might suggest different underlying pathogeneses for this overlapping condition. No robust study has definitively demonstrated the sequence of diseases in RA/SS.

We aimed in this study to describe the baseline characteristics and risk factors of patients with RA/SS in a tertiary medical centre in China. Based on the comparative results of RA/SS with RA or pSS controls, risk factors for RA developing SS and SS developing RA were further identified. Finally, patients with SS-onset RA, RA-onset SS, and simultaneous RA and SS were analysed and compared.

## Patients and Methods

### Patients and controls

We performed a retrospective study of patients with overlapping RA and SS (RA/SS) in Peking Union Medical College Hospital (PUMCH) in China from January 2012 to January 2017. The gender- and age-matched patients with pSS and patients with RA during the same period were randomly chosen as controls with a ratio of 2:1. The diagnosis of RA was according to the criteria developed and validated by the American College of Rheumatology (previously the American Rheumatism Association) in 1987^8^. The diagnosis of SS was based on the revised version of the diagnostic criteria of the American-European Consensus Group^1^. The exclusion criteria were patients diagnosed with other connective tissue diseases, including systemic lupus erythaematosus (SLE), systemic scleroderma, idiopathic inflammatory myositis and mixed connective tissue disease. Patients with overlapping RA and SS (overlap RA/SS) were defined as patients who fulfilled the criteria of both RA and SS.

This study was approved by the Institutional Review Board (IRB) of PUMCH, and all methods were performed in accordance with the relevant guidelines and regulations. All patients provided written informed consent before enrollment.

### Data collection

Data on demographic features and clinical and laboratory findings were retrospectively collected. Clinical features, including xerostomia, xeophthalmia, swollen joint count (SJC), and tender joint count (TJC), were evaluated. Visceral lesions, including interstitial lung disease (ILD) and haematological, nervous, and renal involvements, were systematically reviewed. Laboratory findings were obtained from individual patients when they first visited our institution. Positivity for serum anti-CCP (≥25 U/ml) and RF (≥15 IU/ml) in these patients was determined with an enzyme-linked immunosorbent assay (ELISA) using a specific kit (Euroimmun, Lubeck, Germany) and the nephelometry method (Behring, Germany), respectively. Screening for autoantibodies to SSA and SSB was systematically performed using Ouchterlony double-gel immunodiffusion and Western blotting. All of the tests were performed at the clinical rheumatology immunology laboratory at PUMCH. Radiographic investigations, including posterior–anterior X-radiographs and/or magnetic resonance imaging (MRI) of the hands were used to evaluate the joint lesions in these patients. And all the evaluations of images were performed by two radiographic specialists. All data are available.

### Statistical analyses

The Statistical Package for the Social Sciences (SPSS), version 16.0 (SPSS Inc., Chicago, IL, USA), was used to perform the data analysis. The descriptive data are reported as means and standard deviations, medians and interquartile ranges, or frequencies (percentages). The χ^2^ test, Fisher’s exact test, the Mann-Whitney U-test, and Student’s *t*-test were performed as appropriate. We compared three groups using a one-way ANOVA or Kruskal-Wallis test for continuous variables and a χ^2^ test for categorical variables. Univariate and forward stepwise multivariate Cox proportional regression analyses were performed to identify risk factors. Entry and removal probabilities for stepwise regressions were 0.05 and 0.1, respectively. For all analyses, the probability values were two-sided and P < 0.05 was considered to be significant.

## Results

### Study population

A total of 105 consecutive patients with RA/SS who met all inclusion and exclusion criteria were enrolled in the study. Baseline demographic and clinical characteristics of all patients are shown in Table 1. Ninety-seven (92.4%) patients were female. The mean age at diagnosis of SS and RA was 51.5 ± 13.3 years, and 45.2 ± 14.7 years, respectively. The median disease duration was 47.6 ± 37.3 months. Sixty-eight (64.8%) of the overlap patients had onset of RA, 27 (25.7%) patients had simultaneous onset RA/SS, and 10 (9.5%) patients had onset of SS. Xerostomia and xeophthalmia were detected in 81.0% and 69.5% of the RA/SS patients, respectively. All patients with RA/SS had arthritis, with mean counts of 17.8 ± 10.2 swollen joints and 18.0 ± 10.2 tender joints. Symmetric arthritis, hand arthritis and bone erosion were observed in 99.0%, 98.1% and 100% of these patients, respectively 86 patients with RA/SS had erosion of the joints in posterior–anterior X-radiographs of the hands, while 19 patients with RA/SS showed joint surface destruction in MRI of the hands. Based on thoracic high-resolution computed tomography (HRCR) findings, 74 (70.5%) patients had evidence of interstitial lung disease (ILD). Forty-five (42.8%) patients had haematologic involvement, including anaemia (30, 28.6%), leukopenia (12, 11.4%) and thrombocytopenia (7, 6.7%). The positivity of anti-SSA and anti-SSB was 70.5% and 23.8%, respectively. RF and anti-CCP were detected in 88.6% and 76.6% of these patients.

**Table 1:** Characteristics of the 105 patients with RA/SS.

	All patients with RA/SS (n = 105)	Patients with RA-onset (n = 68)	Patients with simultaneous RA/SS (n = 27)	Patients with SS-onset (n = 10)
Demographic features				
Female, (n, %)	97 (92.4)	61 (89.7)	26 (96.3)	10 (100)
Age at diagnosis of SS (year, mean ± SD)	51.5 ± 13.3	52.8 ± 12.8	51.0 ± 12.7	44.6 ± 16.9
Age at diagnosis of RA * (year, mean ± SD)	45.2 ± 14.7	42.2 ± 17.8	51.0 ± 12.7	49.7 ± 14.6
Time interval (months, median, IQR)	47.6 ± 27.3	234.6 ± 102.2	0	34.8 ± 17.0
Symptoms				
Xerostomia (n, %)	85 (81.0)	54 (79.4)	23 (85.2)	8 (80.0)
Xeophthalmia (n, %)	73 (69.5)	48 (70.6)	19 (70.4)	6 (60.0)
Fever* (n, %)	14 (13.3)	13 (19.1)	0 (0)	1 (10.0)
Rash (n, %)	4 (3.8)	1 (1.5)	2 (7.4)	1 (10.0)
SJC (number, mean ± SD)	17.8 ± 10.2	18.7 ± 9.9	17.1 ± 10.0	16.1 ± 11.8
TJC (number, mean ± SD)	18.0 ± 10.2	18.7 ± 9.8	17.1 ± 10.0	16.1 ± 11.8
ILD (n, %)	74 (70.5)	49 (72.1)	18 (66.7)	7 (70.0)
Anaemia (n, %)	30 (28.6)	19 (27.9)	9 (33.3)	2 (20.0)
Leukopenia (n, %)	12 (11.4)	9 (13.2)	3 (11.1)	0 (0.0)
Thrombocytopenia* (n, %)	7 (6.7)	7 (10.3)	0 (0)	0 (0.0)
Nervous system involvement (n, %)	9 (8.6)	6 (8.8)	0 (0)	1 (10.0)
Renal involvement (n, %)	15 (14.3)	10 (14.7)	2 (7.4)	3 (30.0)
Laboratory findings (n, %)				
Elevated ESR (n, %)	78 (74.3)	55 (80.9)	16 (59.3)	7 (70.0)
Elevated CRP (n, %)	64 (61.0)	43 (63.2)	16 (59.3)	5 (50.0)
Elevated immunoglobulins (n, %)	55/87 (63.2)	36/57 (63.2)	15/22 (68.2)	4/8 (50.0)
ANA( + ) (n, %)	85 (81.0)	57 (83.8)	20 (74.1)	8 (80.0)
Anti-Ro/SSA( + ) (n, %)	74 (70.5)	48 (70.6)	18 (66.7)	8 (80.0)
Anti-La/SSB( + ) (n, %)	25 (23.8)	15 (22.1)	6 (22.2)	4 (40.0)
RF( + ) (n, %)	93 (88.6)	59 (86.8)	25 (92.6)	9 (90.0)
Anti-CCP( + ) (n, %)	72 (76.6)	47/64 (73.4)	17/21 (81.0)	8/9 (88.9)
Objective xerostomia (n, %)	70/80 (87.5)	46/54 (85.2)	18/19 (94.7)	6/7 (85.7)
Positive ocular tests (n, %)	79/84 (94.0)	53/57 (93.0)	19/19 (100)	7/8 (87.5)
Treatment				
Use of glucocorticoids (n, %)	89 (84.8)	60 (88.2)	21 (77.8)	8 (80.0)
Use of immunosuppressant (n, %)	100 (95.2)	64 (94.1)	27 (100)	9 (90.0)

RA: Rheumatoid arthritis; SS: Sjogren syndrome; ILD: Interstitial lung disease; ESR: Erythrocyte sedimentation rate; CRP: C-reactive protein; ANA: Anti-nuclear antibody; Anti-Ro/SSA: Anti-Ro/SSA antibody; Anti-La/SSB: Anti-La/SSB antibody; RF: Rheumatoid factor; Anti-CCP: Anti-cyclic peptide containing citrullin antibody;

*comparing among the three subgroups of patients with RA onset, simultaneous RA/SS, and SS-onset, p < 0.05.

### Clinical features of subgroups associated with disease sequence

Comparisons of demographic and clinical characteristics were made among the subgroups of RA-onset, simultaneous RA/SS, and SS-onset patients. The results showed that the three subgroups shared most of the features similarly. Significant differences were found for age at RA diagnosis (p = 0.016), fever (p = 0.008) and thrombocytopenia (p = 0.042) among the three subgroups. When all pairs of groups were compared, patients with RA-onset SS were diagnosed with RA at an earlier age (42.2 ± 17.8 year) than patients with simultaneous RA/SS (51.0 ± 12.7 year) (p = 0.007). Fever was found more frequently in patients with RA-onset SS (19.1%) than in patients with simultaneous RA/SS (0%) (p = 0.014). No significant differences were identified for thrombocytopenia between any pair of groups (p > 0.05) (Table 1).

### Comparison of patients with RA/SS versus controls

The clinical features, biologic features, and treatment were compared between RA/SS patients and age and gender matched RA controls or pSS controls (shown in Table 2). Compared to pSS controls, RA/SS patients were more likely to have arthritis (100% vs 8.3%, p < 0.001), ILD (70.5% vs 29.0%, p < 0.001) and anaemia (28.6% vs 9.5%, p < 0.001). A higher prevalence of ESR (74.3% vs 55.2%, p < 0.001), CRP (61.0% vs 39.5%, p < 0.001), RF (88.6% vs 42.9%, p < 0.001) and anti-CCP (76.6% vs 25.0%, p < 0.001) was detected in RA/SS than pSS controls. More patients with RA/SS received treatment of corticosteroids (84.8% vs 60.7%, p < 0.001) and immunosuppressants (95.2% vs 28.6%, p < 0.001) than pSS controls.

**Table 2:** Comparisons of characteristics in patients with RA/SS vs controls.

	RA/SS cases (n = 105)	pSS controls (n = 210)	RA controls (n = 210)	P value (RA/SS vs pSS)	P value (RA/SS vs RA)
Clinical features					
Xerostomia (n, %)	85 (81.0)	155 (73.8)	62 (29.5)	0.139	<0.001
Xeophthalmia (n, %)	73 (69.5)	127 (60.5)	45 (21.4)	0.119	<0.001
Fever (n, %)	14 (13.3)	28 (13.3)	49 (23.3)	0.978	0.036
Rash (n, %)	4 (3.8)	15 (7.1)	7 (3.3)	0.249	0.808
Arthritis (n, %)	105 (100)	15/180 (8.3)	210 (100)	<0.001	1.000
Swollen joint count (mean ± SD)	17.8 ± 10.2	1.2. ± 0.6	15.8 ± 7.3	<0.001	0.979
Tender joint count (mean ± SD)	18.0 ± 10.2	3.1 ± 0.9	16.7 ± 7.5	<0.001	0.985
Symmetric arthritis (n, %)	104 (99.0)	34 (16.2)	209 (99.5)	<0.001	1.000
Hand arthritis (n, %)	103 (98.1)	36 (17.1)	207 (98.6)	<0.001	1.000
Erosion of joints (n, %)	105 (100)	0 (0)	210 (100)	<0.001	1.000
Interstitial lung disease (n, %)	74 (70.5)	61 (29.0)	46 (21.9)	<0.001	<0.001
Haematologic involvement (n, %)	45 (42.8%)	78 (37.1%)	43 (20.4%)	0.330	<0.001
Anaemia (n, %)	30 (28.6)	20 (9.5)	40 (19.0)	<0.001	0.062
Leukopenia (n, %)	12 (11.4)	68 (32.4)	4 (1.9)	<0.001	<0.001
Thrombocytopenia (n, %)	7 (6.7)	16 (7.6)	5 (2.4)	0.700	0.061
Nervous system involvement (n, %)	9 (8.6)	7 (3.3)	8 (3.8)	0.058	0.078
Renal involvement (n, %)	15 (14.3)	24 (11.4)	25 (11.9)	0.412	0.550
Biologic features					
Elevated ESR (n, %)	78 (74.3)	116 (55.2)	165 (78.6)	0.001	0.393
Elevated CRP (n, %)	64 (61.0)	83 (39.5)	181 (86.2)	<0.001	<0.001
Elevated immunoglobulins (n, %)	55/87 (63.2)	119 (56.7)	137 (65.2)	0.284	0.740
ANA, n (%)	85 (81.0)	181 (86.2)	107 (51.0)	0.238	<0.001
Anti-Ro/SSA (n, %)	74 (70.5)	127 (60.5)	15 (7.1)	0.084	<0.001
Anti-La/SSB (n, %)	25 (23.8)	50 (23.8)	6 (2.9)	0.959	<0.001
RF (n, %)	93 (88.6)	90 (42.9)	168 (79.0)	<0.001	0.057
Anti-CCP( + ) (n, %)	72/94 (76.6)	9/36 (25.0)	173 (82.3)	<0.001	0.104
Treatment					
Use of corticosteroid (n, %)	89 (84.8)	147 (60.7)	29 (13.8%)	<0.001	<0.001
Use of immunosuppressant (n, %)	100 (95.2)	60 (28.6)	206 (98.1%)	<0.001	0.166

RA: Rheumatoid arthritis; SS: Sjogren syndrome; ILD: Interstitial lung disease; ESR: Erythrocyte sedimentation rate; CRP: C-reactive protein; ANA: Anti-nuclear antibody; Anti-Ro/SSA: Anti-Ro/SSA antibody; Anti-La/SSB: Anti-La/SSB antibody; RF: Rheumatoid factor; Anti-CCP: Anti-cyclic peptide containing citrullin antibody.

**Figure 1:**
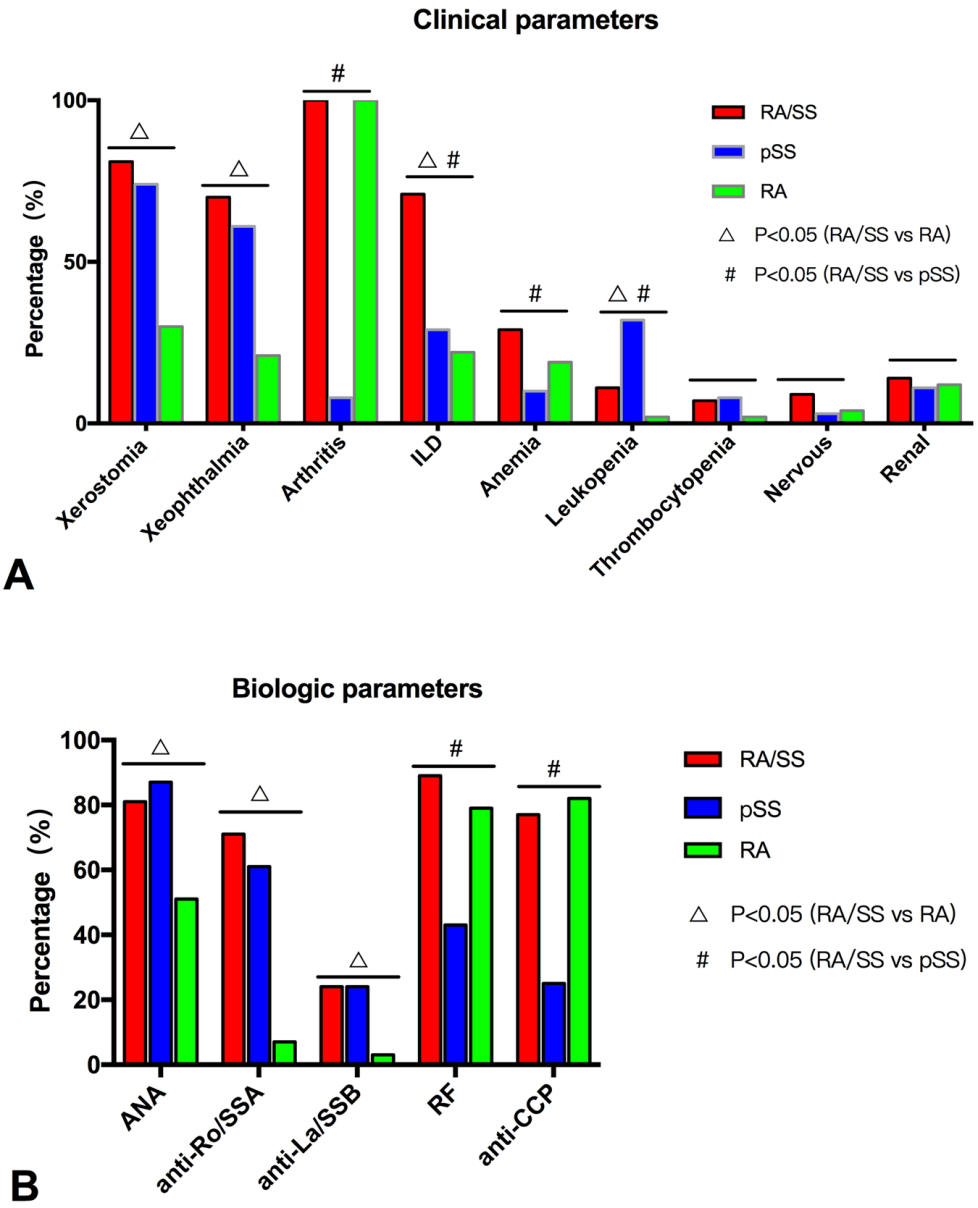
The candidate clinical and biologic parameters for the overlap diseases. (**A**) Comparisons of clinical parameters among patients with RA/SS and controls. (**B**) Comparisons of biologic parameters among patients with RA/SS and controls. RA: Rheumatoid arthritis; SS: Sjogren syndrome; ILD: Interstitial lung disease; ANA: Anti-nuclear antibody; Anti-Ro/SSA: Anti-Ro/SSA antibody; Anti-La/SSB: Anti-La/SSB antibody; RF: Rheumatoid factor; Anti-CCP: Anti-cyclic peptide containing citrullin antibody.

Compared to RA controls, the overlap patients presented with more xerostomia (81.0% vs 29.5%, p < 0.001), xeophthalmia (69.5% vs 21.4%, p < 0.001), ILD (70.5% vs 21.9%, p < 0.001) and leucopenia (11.4% vs 1.9%, p < 0.001). In laboratory findings, the positivity of ANA (81.0% vs 51.0%, p < 0.001), anti-SSA (70.5% vs 7.1%, p < 0.001) and anti-SSB (23.8% vs 2.9%, p < 0.001) were significantly higher in RA/SS patients than in RA controls. More patients with RA/SS received treatment with corticosteroids than RA controls (84.8% vs 13.8%, p < 0.001). There were no significant differences in the frequency of use of immunosuppressants (p > 0.05). Figure 1 depicts the candidate clinical and biologic parameters for the overlap diseases.

### Risk factors for patients with RA or SS to develop RA/SS

As shown in Table 3, univariate analysis identified arthritis (OR = 92.076, 95%CI 21.325–397.553, p < 0.001), anaemia (OR = 4.072, 95%CI 2.244–7.389, p < 0.001), RF (OR = 10.532, 95%CI 5.077–21.847, p < 0.001) and anti-CCP (OR = 11.232, 95%CI 5.750–21.941, p < 0.001) as potential risk factors for SS to develop RA. In contrast, ILD was not a potential risk factor (p > 0.05). The results of multivariate analysis confirmed that arthritis (OR = 44.804, 95%CI 9.405-21.434, p < 0.001), RF (OR = 5.973, 95%CI 2.228–16.016, p < 0.001), and anti-CCP (OR = 2.545, 95%CI 1.067–6.068, p = 0.035) were independent risk factors for SS developing RA.

**Table 3:** Risk factors for patients with SS or RA to develop RA/SS.

	Variables	Univariate analysis		Multivariate analysis	
		Univariate HR (95% Cl)	P-value	Multivariate HR (95%Cl)	P-value
SS develop RA	**Arthritis**	92.076 (21.325–397.553)	<0.001	44.804 (9.405–21.434)	<0.001
	ILD	1.644 (0.844–3.203)	0.144	/	/
	**Anaemia**	4.072 (2.244–7.389)	<0.001	/	>0.05
	**RF**	10.532 (5.077–21.847)	<0.001	5.973 (2.228–16.016)	<0.001
	**Anti-CCP**	11.232 (5.750–1.941)	<0.001	2.545 (1.067–6.068)	0.035
RA develop SS	**Xerostomia**	11.591 (4.977–26.992)	<0.001	3.960 (1.110–14.122)	0.034
	Xeophthalmia	8.111 (3.473–18.944)	<0.001	/	>0.05
	**ILD**	9.847 (4.089–24.713)	<0.001	6.210 (1.903–20.270)	0.002
	Leukocytopenia	5.161 (0.649-41.041)	0.121	/	/
	**Anti-SSA**	30.237 (8.681–105.321)	<0.001	24.640 (6.228–97.481)	<0.001

SS: Sjogren syndrome; RA: Rheumatoid arthritis; OR: Odds ratio; CI: Confidence interval; ILD: Interstitial lung disease; RF: Rheumatoid factor.

As for potential risk factors for RA to develop SS, univariate analysis showed xerostomia (OR = 11.591, 95%CI 4.977–26.992, p < 0.001), xeophthalmia (OR = 8.111, 95%CI 3.473–18.944, p < 0.001), ILD (OR = 9.847, 95%CI 4.089–24.713, p < 0.001), and anti-SSA (OR = 30.237, 95%CI 8.681–105.321, p < 0.001) were significant variables. However, leukopenia was not significant (p > 0.05). Multivariate analysis confirmed that xerostomia (OR = 3.960, 95%CI 1.110–14.122, p = 0.034), ILD (OR = 6.210, 95%CI 1.903–20.270, p = 0.002), and anti-SSA (OR = 24.640, 95%CI 6.228–97.481, p < 0.001) were independent predictors of RA developing SS.

## Discussion

This study is one of the largest case controlled studies of patients with RA/SS with well-defined clinical and laboratory features, focusing on the identification of risk factors and comparisons of the chronologic orders. In this study, 105 consecutive patients with RA/SS in the last 5 years in our centre were enrolled. Most of them were female with typical erosive arthritis, Sicca symptom, and visceral involvements. Arthritis, RF and anti-CCP were risk factors for SS developing RA, while ILD, xerostomia and anti-SSA were risk factors for RA to be accompanied by SS. The RA-onset, SS-onset and simultaneous onset subgroups of the RA/SS patients shared most of the clinical features similarly.

Whether RA/SS is a distinct entity with overlapping RA and SS or is a subset of RA is a subject of debate. In this study, most RA/SS patients had onset of RA first (64.8%), while 9.5% of patients had initial onset of SS. These findings were consistent with previous reports^4^. However, different from RA, the RA/SS patients had prominent visceral organ involvement, including ILD and hematologic involvements. These features were consistent with a previous report^9^. The coexistence of the extra-articular/glandular manifestations would behave as a bad prognostic factor, and partially explain the greater need for glucocorticoids in this subgroup of patients. Of note, this study demonstrated that the RA-onset SS patients were diagnosed with RA at an early age (42.2 years old) and developed SS within a median of 234.6 months. This result was similar to previous studies that indicated a slowly progressive disease course for RA to develop SS^4^.

To the best of our knowledge, very few data are available concerning the risk factors for RA/SS. The present study highlights the clinical significance of arthritis (OR = 44.804). Approximately 50 percent of patients with primary SS complain of joint pain, with or without evidence of arthritis^10,11^. The arthropathy in SS is usually asymmetric, intermittent, typically nonerosive and nondeforming^11^. We observed that when symmetrical multiple swelling and tenderness are present, the patients should be considered to have an overlap with RA. The application of ultrasound imaging, X-ray or MRI of the joints enabled the early diagnosis of bone erosion and might be a great help in the detection of early RA overlapping in SS patients^11^. The anti-CCP antibody is highly sensitive and specific for the diagnosis of RA and is associated with bone erosion^12^. The incidence of RF and anti-CCP in SS is 42.9% and 25.0% in our Chinese pSS patients, which is similar to other studies on Chinese patients^4,10,13^. SS patients with such indications should be recognized and aggressively treated to control joint inflammation.

ILD is the most common extra-articular manifestation of RA in the lung, with a prevalence of 19–44% in RA patients^14,15^. Similarly, among a variety of conditions affecting the organs in pSS, ILD is one of the most common extraglandular complications, with a prevalence varying from 9% to 75%^16,17^. In the present study, we found a prominent high prevalence of ILD in patients with RA/SS. Additionally, ILD was identified as a strong indicator for RA developing SS, which was not previously reported and should arouse clinicians’ attention. Because patients in combination with ILD tend to receive more vigorous GC treatment and have a poor prognosis, investigations such as HRCT manifestation and pulmonary function tests with a detailed evaluation of ILD in RA/SS should be addressed in the future. The prevalence of Sicca symptoms varies from 11.4% to 60.7% in RA^7^. In our study group, 29.5% and 21.4% of the patients in the control group (RA without coexisting SS) had xerostomia and xeophthalmia, respectively. Xerostomia was further detected as a risk factor for RA to develop SS, indicating the importance of monitoring Sicca symptoms in association with overlapping SS. Anti-SSA antibody is associated with an extensive spectrum of autoimmune diseases, such as pSS, SS/SLE overlap, SLE, neonatal lupus and primary biliary cirrhosis^18^. Additionally, in this study, RA patients with positive anti-SSA were more prone to overlap with SS, and there should be objective investigations to identify whether they have overlapping diseases.

Currently we lack evidence-based recommendations for the follow-up and management of patients with RA/SS. The implication of the therapeutic strategy should be further discussed. And it would be necessary to define recommendations by multidisciplinary groups to these concerns. According to the results for risk factors, we recommend the screening of anti-CCP to patients with SS and anti-SSA/Ro to patients with RA for the overlap RA&SS. SS has been reported in association with a large variety of autoimmune diseases including RA, systemic lupus erythematosus, systemic scleroderma, autoimmune hepatitis, and primary biliary cirrhosis, and the like. Thus, we suggest patients with SS perform active search for a second or more autoimmune disease. All the autoimmune diseases share common phenotype and mechanism. Genetic and environmental factors might influence the development of the polyautoimmunity disease^19^. There are also some limitations of this study. First, we could not exclude the possibility of selection bias. Since PUMCH is the tertiary referral centre for complicated patients nationwide, the enrolled patients and controls were more likely to have active diseases and comorbidities. Second, this was a retrospective study; thus, patients with risk factors need to be further carefully followed-up after developing RA or SS. Third, lung physiology and radiology (especially HRCT imaging manifestations) of RA/SS-related ILD were not adequately described in the present study.

In conclusion, RA/SS is a special overlap syndrome of RA and SS, which is characteristically manifested with more visceral involvements, including ILD and haematologic manifestations. When in combination with arthritis/positive RF/anti-CCP, patients with pSS should be aware of erosive arthritis. When RA patients develop xerostomia/ILD/anti-SSA, they should be encouraged to have an examination for SS. The proper recognition and prompt early diagnosis of RA/SS is important when choosing suitable therapies and improving a patient’s prognosis.
